# The viability gambit: an optimized disinfection protocol for industrial hemp (*Cannabis sativa* L.) seeds

**DOI:** 10.3389/fpls.2026.1907649

**Published:** 2026-10-13

**Authors:** Rachna Gowlikar, Grace Pender, Joanna Kacprzyk, Alice Destailleur, Aditya Nayak, Rainer Melzer, Susanne Schilling

**Affiliations:** 1 School of Biology and Environmental Science and UCD Earth Institute, University College, Dublin, Ireland; 2 Plantik Biosciences, Paris, France

**Keywords:** *Cannabis sativa* L., hemp, hydrogen peroxide, hypocotyl regeneration, octave, seed disinfection, sodium hypochlorite, tissue culture

## Abstract

Hemp (*Cannabis sativa* L.) is an increasingly important crop with applications spanning fiber, seed oil and bioactive cannabinoid production, yet the development of reliable tissue culture systems for this species remains a significant challenge. The establishment of axenic seedling cultures is a prerequisite for hypocotyl-based regeneration and future genetic transformation pipelines, but hemp seeds can harbor diverse microbial communities which can make seed disinfection challenging. Here, we evaluate several disinfection strategies across seven industrial hemp cultivars including treatment with hydrogen peroxide and sodium hypochlorite as well as the Prochloraz-based fungicide Octave and Plant Preservative Mixture (PPM™) on both, in-house produced and commercially sourced seed material. Across all treatments and accessions, in-house glasshouse-harvested seeds achieved higher disinfection rates than commercially sourced material regardless of the protocol applied (n=3681 and 1886, respectively). Among the disinfection treatments evaluated, a baseline of 75% ethanol combined with sequential hydrogen peroxide or sodium hypochlorite incubation performed well for certain seed batches, though variation in viable seedling rate remained high (~30% to ~80% viable seedlings). Supplementation with PPM™ increased the rates of viable seedlings to ~60% in some cases. Beyond their effect on contamination, disinfection treatments appeared to influence the morphogenic fate of hypocotyl explants independently of disinfection outcomes. Here, seedling treatment with Octave was associated with shoot and root co-regeneration in the absence of exogenous plant growth regulators. Together, these findings provide a practical framework for axenic hemp seed culture that can be used as starting point requiring local adaptation based on seed source, batch history and the intended downstream application.

## Introduction

Hemp (*Cannabis sativa* L.) is a highly versatile crop with a broad range of industrial, agricultural and pharmacological applications, including the production of fiber, seed oil and bioactive cannabinoids such as cannabidiol (CBD) (Small, 2015; Andre et al., 2016; Schilling et al., 2023). Following decades of regulatory restriction, hemp cultivation has undergone a global resurgence and is now practiced across more than 30 countries. This expansion has created a growing demand for scalable, genetically consistent planting material and has renewed interest in developing robust tissue culture systems for this species (Chandra et al., 2017).

Tissue culture and plant regeneration are foundational tools in modern crop research and breeding. The ability to regenerate whole plants from isolated explants is a prerequisite for genetic transformation, enabling the stable introduction of defined traits and the functional characterization of genes of interest. In many crop species, efficient transformation and regeneration pipelines have accelerated both basic research and the development of improved varieties (Hesami et al., 2021). In hemp, however, these tools remain relatively poorly developed, representing a significant bottleneck for both fundamental research and applied breeding programs. High levels of recalcitrance are reported for *in vitro* shoot regeneration of hemp (Wielgus et al., 2008; Smýkalová et al., 2019; Adhikary et al., 2021), making transformation based on tissue culture challenging. In recent years, hypocotyl explants are among the most promising target tissues for hemp tissue culture and transformation, as they consist of rapidly dividing cells with broad developmental plasticity and have been shown to undergo organogenesis under hormone-free conditions in some accessions (Galán-Ávila et al., 2020; Hesami et al., 2021). However, the utility of hypocotyl-based regeneration systems depends entirely on the successful axenic establishment of seedlings from surface-sterilized seeds. This upstream step is non-trivial in hemp. Seeds harbor a taxonomically diverse endophytic microbiome, including bacterial genera such as *Pseudomonas*, *Pantoea* and *Bacillus*, and fungal endophytes from genera including *Alternaria*, *Cladosporium* and *Aureobasidium*, which are deeply embedded within seed tissues and resistant to standard surface disinfection (Punja et al., 2018). *In vitro* cultures are additionally susceptible to fast-growing opportunistic pathogens common in hemp production environments, including *Botrytis cinerea* and *Penicillium* spp (Punja et al., 2019). Previous studies have established hemp seed disinfection with hydrogen peroxide (Sorokin et al., 2021) as well as ethanol in combination with sodium hypochlorite (Galán-Ávila et al., 2020; Hesami et al., 2021). However, systematic comparisons of the different disinfection protocols remain scarce. A further complication is the substantial genetic and phenotypic diversity among industrial hemp cultivars (Vergara et al., 2017; Kavanagh et al., 2025; Trubanová et al., 2026). Seed provenance adds another layer of variability: field-harvested commercial seeds are exposed to diverse soil and airborne microbial communities during development, harvest and storage, whereas seeds produced under controlled glasshouse conditions carry a lower and more predictable contamination burden. These sources of variation are rarely accounted for systematically in published disinfection protocols, which are typically reported for a single cultivar and seed source without broader validation.

Here, we present a comparative analysis of seed surface disinfection protocols across seven industrial hemp accessions, evaluating standalone chemical treatments including H_2_O_2_ and NaOCl at varying concentrations alongside combinatorial protocols using a standard ethanol and sodium hypochlorite baseline supplemented with the Prochloraz-based fungicide Octave and Plant Preservative Mixture™ (PPM™). We further assess the regeneration competence of hypocotyl explants in hormone-free culture. Our results illustrate the batch- and context-dependent nature of disinfection outcomes and support the development of more robust regeneration systems for this increasingly important crop.

## Materials and methods

### Plant material and cultivation

Hemp seeds from seven cultivars (‘Felina 32’, ‘Ferimon’, ‘Santhica 27’, ‘Kompolti’, ‘USO31’, ‘Futura 75’ and ‘Fedora 17’) were obtained from two sources: commercially certified field-harvested seed lots (HEMPOINT, Czech Republic) and in-house produced seed stocks (**Supplementary Table S1**). In-house seeds of were generated from plants cultivated under controlled glasshouse conditions at University College Dublin, maintained under glasshouse conditions following (Schilling et al., 2023). All seeds were stored at 4 °C in silica gel in darkness until use. Hemp explants on were grown on ½ MS medium in a plant cultivation room under red–blue LEDs, light intensity approximately 500 μmol sec−1 at approximately 15 cm distance from the light source; HORTILED MULTI, 44 W; HORTILUX, https://www.hortilux.com lights at a constant temperature of 25 °C under a 16-hour photoperiod.

### Surface disinfection

All disinfection treatments were preceded by an ethanol incubation in which approximately 30 seeds were agitated at 72 rpm in 40 ml 75% ethanol for three minutes. All disinfection treatments were followed by three to four washing steps. For washing, all liquid was removed completely, and seeds were rinsed with 40 ml sterile distilled water for 20 minutes at 72 rpm each time. In case any lathering was still present washing continued until the discarded liquid was free of any traces of foam formation.

For water, vinegar (1%) and sodium chloride (1%) treatment, 20 seeds were agitated in 30 ml of the respective solution for 15 minutes only adapted the use of vinegar and sodium chloride similar to what has been previously described (Nakaune et al., 2012; Gilbert et al., 2023). For sodium hypochlorite treatments approximately 30 seeds were agitated in 40 ml 20% or 50% commercial bleach (approximately 1% and 3% NaOCl) at 72 rpm for 20 minutes for the hydrogen peroxide treatment, 30 seeds were soaked in 40 ml 1% or 3% H_2_O_2_ in darkness at 25 °C in sequential incubations of 24, 48, 24 hours, similar to what has been described previously (Sorokin et al., 2021). Between each H_2_O_2_ incubation three wash steps with sterile deionized water were conducted. For the Octave treatment, approximately 30 seeds were agitated in a Prochloraz-based fungicide solution (0.8 g Octave (Bayer CropScience) dissolved in 40 ml deionized and autoclaved water) at 180 rpm for two hours, rinsed four to five times with sterile deionized water, then incubated in sterile water in darkness for 48 hours before transfer to growth medium.

Plant Preservative Mixture™ (PPM™, Plant Cell Technology), a broad-spectrum biocide for plant tissue culture, was not applied as a seed treatment but incorporated directly into ½ Murashige and Skoog (MS) basal salt growth medium at the manufacturer’s recommended concentration (0.1%). These treatments were applied individually and in combination in replicates (see **Supplementary Table S1**).

Following surface disinfection, seeds were cultured at a density of 10 seeds per plate in 9 cm diameter Petri dishes containing ½ MS medium and 0.5% agar. The total number of seeds per experiment varied between 20 and 200 (**Supplementary Table S1**). Seeds were scored as germinated after 14 days if the seedling was free from visible contamination and had produced at least one true leaf pair. In case of contamination or if the seedling failed to develop true leaves, seedlings were scored as non-viable.

### *In vitro* regeneration from hemp hypocotyl explants

Hypocotyl explants were excised from sterile seedlings 7–14 days after germination and transferred to hormone-free ½ MS medium (Galán-Ávila et al., 2021). Shoot regeneration was counted as small new leaves emerging; root regeneration was counted as small roots with root hairs emerging. Regeneration was scored after 25 days as “shoot only” if new leaves emerged from the hypocotyl explant, “root only” if regeneration of roots was observed and “shoot and root” if both, roots and shoots developed from the explant. Explants with neither shoot nor root development were scored as “no regeneration”. Explants were transferred to new MS medium every ten to 14 days for approximately five weeks. Some of the explants were transferred to soil after that time and cultivated until flowering (approximately 115 days in total) (Galán-Ávila et al., 2020).

### Statistical analysis and data visualization

All statistical analyses and figures were generated in R (version 2026.04.0; R Core Team, 2026). Two complementary approaches were used depending on the data structure. Seedling disinfection yield was analyzed using binomial generalized linear models (GLM), with pairwise comparisons performed using Tukey’s HSD *post-hoc* test implemented in the R emmeans package (cran.r-project.org/package=emmeans).

Pairwise differences in regeneration outcome were assessed using Fisher’s exact test, which does not rely on large-sample approximations. *Post hoc* tests were conducted using Fisher’s exact. Differences in morphogenic outcome distribution across accessions were assessed using a Chi-squared test of independence with simulated p-values. The overall effect of seed provenance on disinfection rate was tested using a two-sample t-test. Figures were generated using the ggplot2 package (Wickham, 2016).

## Results

### Ethanol combined with hydrogen peroxide or sodium hypochlorite are effective for sterilizing hemp seeds

To establish protocols suitable for routine axenic tissue culture for hemp, seeds of different hemp cultivars were subjected to a variety of treatments with different degrees of stringency and were subsequently germinated on MS agar plates and categorized into viable (not contaminated, developing true leaves) and non-viable (visibly contaminated, no growth of cotyledons or true leaves) (**Figures 1a-c**). Water-only controls and sodium chloride- or vinegar-supplemented seed treatments produced near-universal contamination which resulted in germination failure across all accessions, confirming that more stringent seed treatment is a prerequisite for successful establishment tissue cultures (**Supplementary Figure S1**). Further, treatment using 75% ethanol combined with 1% H_2_O_2_ produced viable disinfected seeds at a ratio of 25 – 75% (**Figure 1d**; **Supplementary Figure S1**), which is lower than the germination rate we observed on soil (42 – 80%; **Supplementary Table S1**).

**Figure 1:**
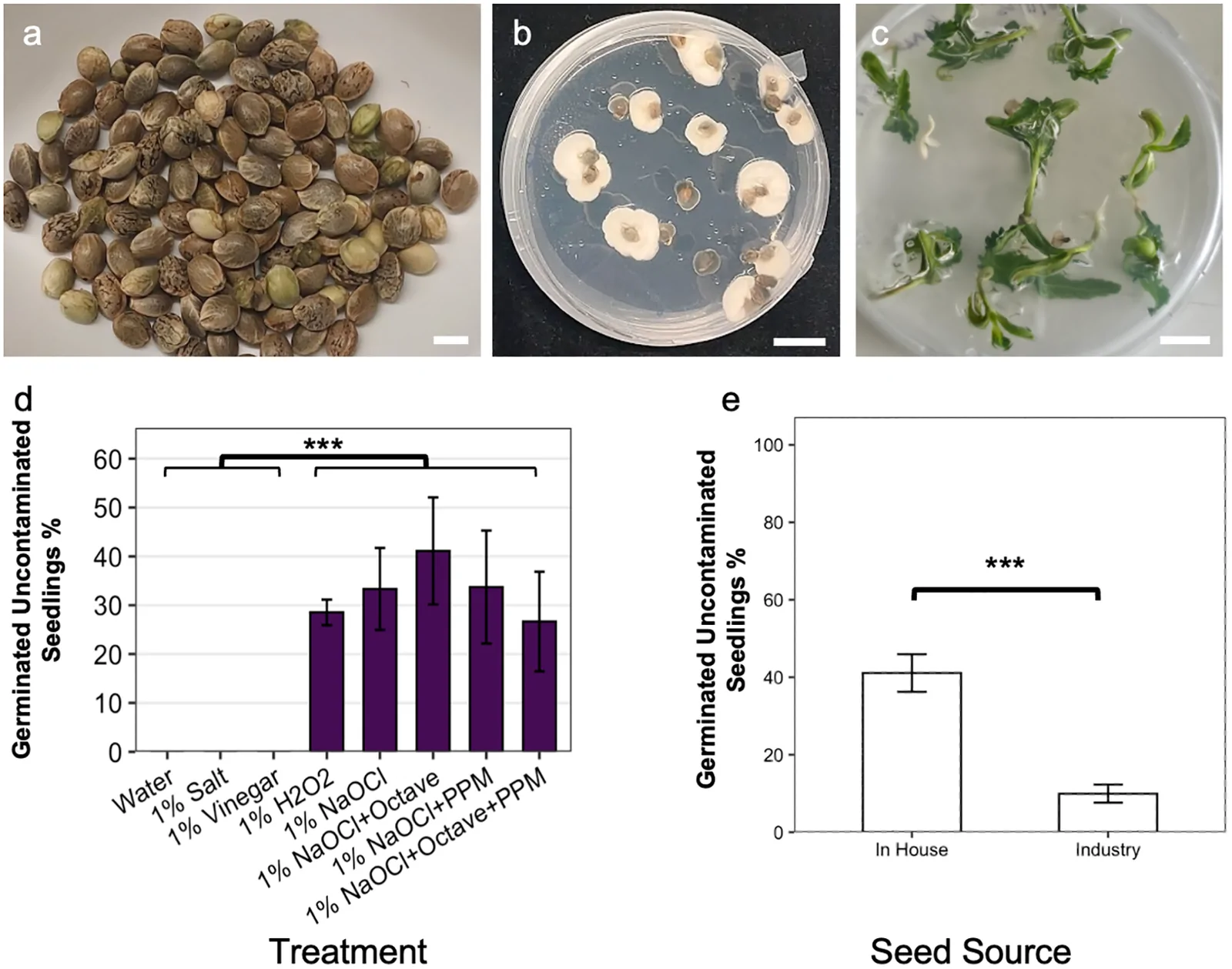
Effect of varying seed disinfection protocols on C*annabis sativa.* L seedling decontamination. Hemp seeds (**a**, untreated hemp seeds) were treated with different disinfection protocols and were scored if they were contaminated **(b)** or disinfected **(c)** after incubation. The mean percentage of disinfected seedlings varied depending on the disinfection protocol used **(d)**, with significant differences between water (n=20), NaCl (sodium chloride) (n=20) or vinegar (n=20) treated plants and all other disinfection methods, including 1% H_2_O_2_ (n=289), 1% NaOCl (n=90), 1% NaOCl combined with Octave (n=90), 1% NaOCl combined with PPM™ (n=90) and 1% NaOCl combined with Octave and PPM™ (n=90) (Tukey’s HSD, ***, p<0.001). Overall, seedlings derived from seeds that were grown in-house (n=3681) showed significantly higher disinfection rates as compared to commercially sourced seeds (‘industry’, n=1886) over a variety of different disinfection treatments (e, ***, p<0.001) (**Supplementary Table S1**). Scale bar 5 mm **(a)** and 10 mm **(b,c)**. Error bars indicate standard error **(d, e)**.

Next, four NaOCl-based protocols were compared to assess whether supplementary biocidal agents improve disinfected seedling yield (**Figure 1d**). In addition to 1% NaOCl alone we supplemented NaOCl with the fungicide Octave and Plant Preservative Mixture (PPM™). Here, the yield of non-contaminated seedlings was significantly higher than in water, sodium chloride and vinegar treatments (Tukey’s HSD, p<0.001), but broadly similar across 1% NaOCl alone, NaOCl combined with Octave, NaOCl combined with PPM™, and NaOCl combined with both Octave and PPM™, with no statistically significant differences detected among the latter treatments (**Figure 1d**). While all experiments outlined so far used seeds produced locally in our greenhouse or growth rooms (termed in-house seeds henceforth), we also tested H_2_O_2_ and NaOCl based protocols on commercially sourced seeds. Commercially sourced seeds generally exhibited lower disinfection rates compared to in-house seeds, yielding an overall mean of ~10% disinfected seedlings across all treatments. (**Supplementary Table S1**). In commercially sourced seeds, NaOCl combined with PPM™ performed significantly better than all other NaOCl based treatments (**Supplementary Figure S2**). The observation that in-house seeds produced a higher percentage of sterile seedlings when compared to industry-sourced seeds persisted across all accessions and treatments in our screens. In-house seeds achieved a higher disinfection rate as compared to commercially sourced material across a variety of different hemp cultivars and treatments in our hands (Student’s t-test, p<0.001; **Figure 1e**; **Supplementary Table S1**). Uncontaminated viable seedlings appeared mostly healthy, free of necrosis or visible signs of stress such as discoloration (**Figure 1c**).

### Disinfection treatments influence hemp hypocotyl regeneration outcomes

One important aspect of tissue culture is plant regeneration. For hemp, regeneration of plants from hypocotyls is one prominent approach. To test hypocotyl regeneration for hemp, hypocotyl explants recovered from sterilized seeds were transferred to hormone-free ½ MS medium and scored for regeneration outcome after 25 days. Notably, we sometimes observed bipolar regeneration from hypocotyl explants, with both shoot and roots developing from opposite sides of the same explant, referred further as root and shoot regeneration. Regeneration of only either shoot or root from hypocotyl explants was also frequently observed (**Figure 2**).

**Figure 2:**
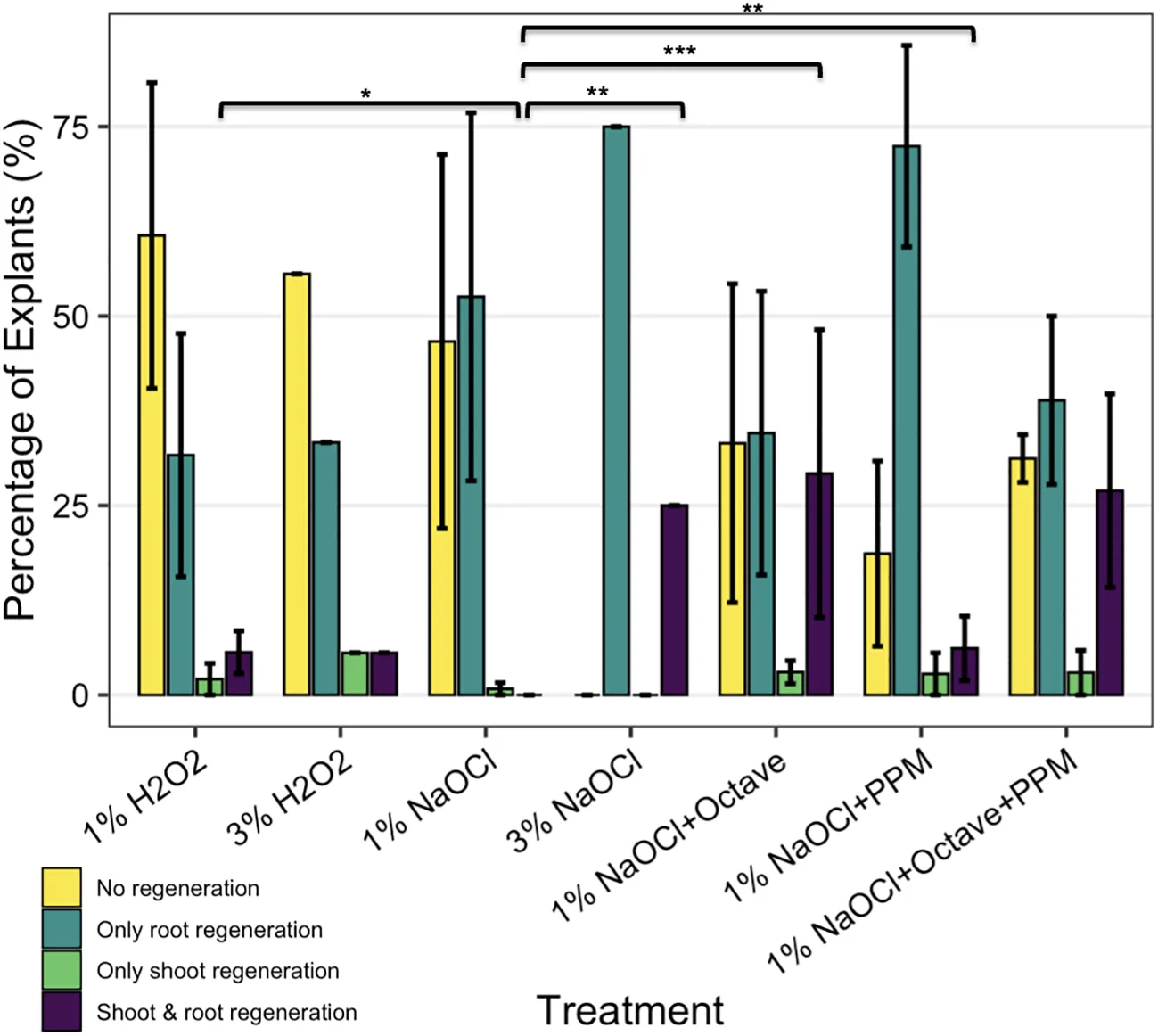
Effect of different disinfection additives on the regeneration outcomes and viability of *Cannabis sativa*. L hypocotyl explants. Explants derived from seedlings of the hemp cultivar ‘Felina 32’ which underwent different disinfection protocols (x-axis) at different times of the year (**Supplementary Table S2**) were scored for their regeneration and categorized as no regeneration (yellow), only root regeneration (teal), only shoot regeneration (green) and shoot and root regeneration (dark blue). Selected significant differences are indicated with brackets (Fishers exact test, **, p<0.01; ***, p<0.001).

Different seed disinfection treatments were analyzed for their effect on hypocotyl regeneration. Most hypocotyl explants generated from seeds of the cultivar ‘Felina 32’ (in-house) disinfected with 1% H_2_O_2_ or NaOCl did not regenerate or regenerated only roots, with only a minor proportion producing shoots or shoots and roots (**Figure 2**). In contrast, explants from seeds disinfected with NaOCl combined with Octave as well as NaOCl combined with Octave and PPM showed a significantly higher proportion of shoot and root regeneration compared to sole NaOCl or H_2_O_2_ treatments (Fisher’s Exact Test, p<0.01). Additionally, the percentage of explants showing no regeneration was significantly lower in explants originally disinfected with 1% NaOCl combined with PPM™ as compared to those treated with 1% NaOCl alone (Fisher’s Exact Test, p<0.001; **Figure 2**; **Supplementary Table S2**). In contrast, explants derived from seeds of the cultivar ‘Ferimon’ (commercial), regenerated shoots as well as roots best when initially disinfected with H_2_O_2_ without the addition of more stringent disinfection agents (**Supplementary Figure S3**).

### Regeneration capacity varied across hemp accessions and was influenced by seed provenance

Given the different responses of the two cultivars tested, we assessed the regeneration of hypocotyl explants across multiple accessions under a standardized disinfection protocol to establish whether regeneration competence is broadly conserved or variable within hemp (**Figure 3**). Explants from four hemp cultivars were evaluated following disinfection with 1% H_2_O_2_ (‘Fedora 17’, ‘Felina 32’, ‘Kompolti’ and ‘USO31’). Significant variation in morphogenic outcome distribution was detected among the accessions (Chi-squared test, χ^2^ = 27.71, df =9, p<0.01). Shoot and root co-regeneration was most frequent in ‘Fedora17’, with around a third of explants, while co-regeneration rate was significantly lower in ‘Kompolti’ and ‘Felina 32’ (Fishers exact test, p<0.05). However, in all cultivars around half of the total explants did not show visible regeneration of either leaves or roots (**Figure 3**).

**Figure 3:**
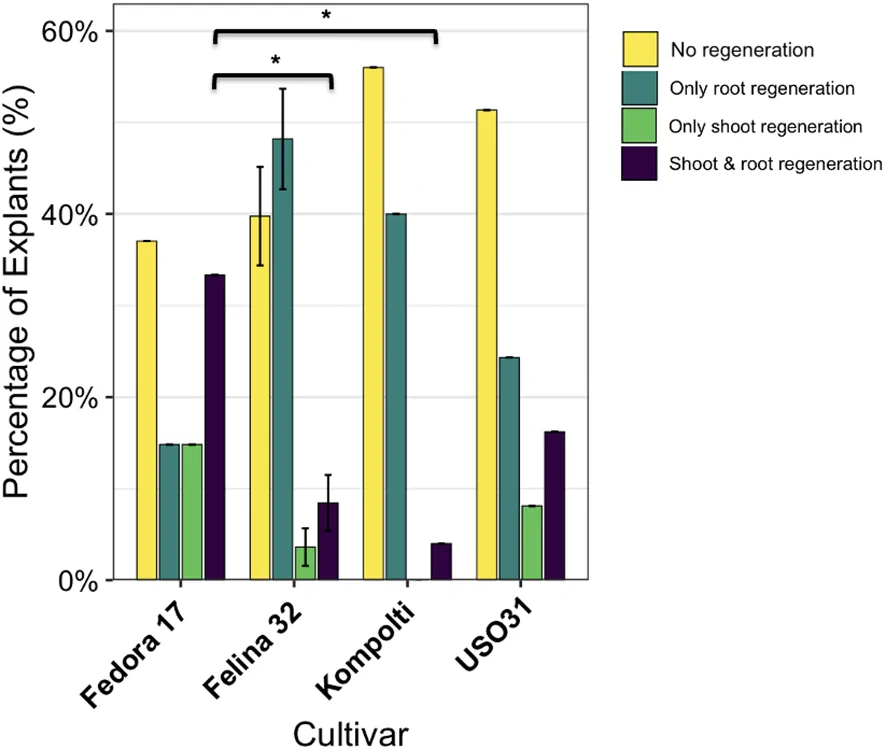
Regeneration outcomes across various *Cannabis sativa.* L cultivars following 1% H_2_O_2_ surface disinfection. Explants of seeds disinfected with 1% H_2_O_2_ of different hemp cultivars were treated at same the time (**Supplementary Table S2**) (‘Fedora 17’ (n=27), ‘Felina 32’ (n=83), ‘Kompolti’ (n=25), ‘USO 31’ (n=37)) were scored for their regeneration and categorized as no regeneration (yellow), only root regeneration (teal), only shoot regeneration (green) and shoot and root regeneration (dark blue). Selected significant differences are indicated with brackets (Fishers exact test, *, p<0.05).

### Seed-to-seed regeneration via hypocotyl explants in hemp

To demonstrate that sterilized explants can complete the full regeneration cycle, the developmental progression from hypocotyl explant to fertile adult plant was documented (**Figure 4**). Sterile hypocotyl explants placed on hormone-free ½ MS medium developed shoots within two weeks (**Figures 4a–d**). Explants regenerated shoots and roots from the same hypocotyl in a polar way, with one end developing shoots and the other developing roots (**Figure 4e**) after approximately three weeks. Plantlets that established both roots and shoots were subsequently transferred to soil and grown to maturity in a growth room (**Figure 4f**). Plants derived from this protocol reached reproductive maturity at 16 weeks and set seed after 19 weeks (**Figures 4g, h**) which were successfully germinated and grown into a mature plant (not shown).

**Figure 4:**
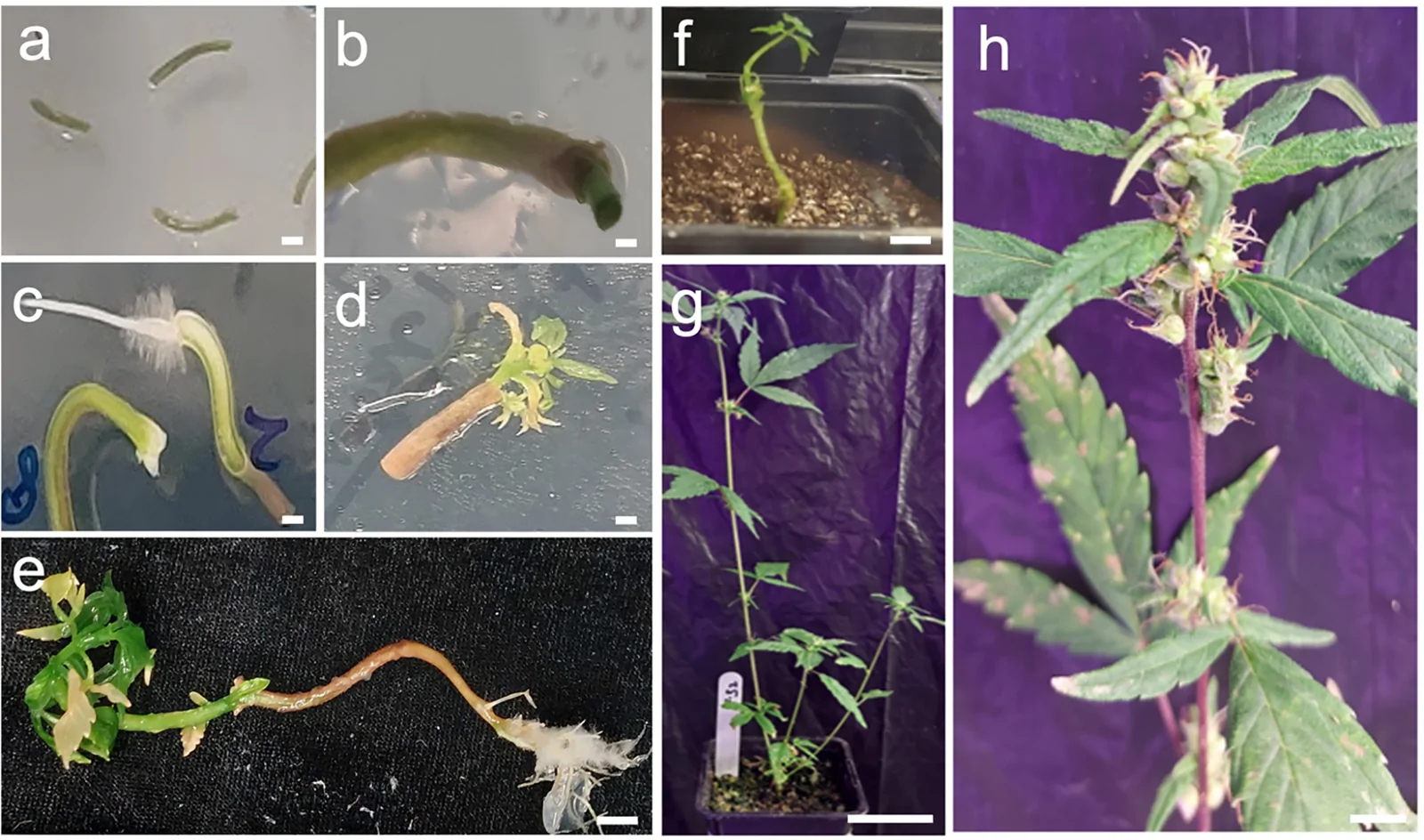
*In vitro* hypocotyl-mediated regeneration and *ex vitro* establishment of *Cannabis sativa.* **(L)** Hypocotyl explants of the hemp cultivar ‘Felina 32’ were excised from aseptically germinated seedlings and inoculated on hormone free growth medium **(a)**. After approximately four days growth of ground tissue was observed **(b)**. Adventitious roots emerged after about five days directly from the hypocotyl explant **(c)**, demonstrating direct root regeneration without an intervening callus phase. Multiple shoots with leaves emerged from the regenerating hypocotyl explant after approximately 7–15 days **(d)**. Both shoots and roots were present on a proportion of explants after a minimum of 25 days **(e)**. Rooted plantlets with shoots were transferred to soil and acclimatized in a vermiculite-soil based substrate under controlled humidity and light conditions **(f)**. Hemp plants reached full maturity and reproductive stage after circa 115 days **(g)** and were able to produce mature seeds after a total of 135 days of cultivation (h, white arrowhead). Scale bars: **(a, e)** = 10 mm; (b–d) = 5 mm; **(f)** = 10 mm; **(g)** = 50 mm; **(h)** = 20 mm.

## Discussion

Establishing axenic seedling cultures from surface-disinfected hemp seeds is a prerequisite for hypocotyl-based regeneration and any downstream genetic transformation pipeline, yet this upstream step has received surprisingly little systematic attention in the hemp tissue culture literature, where usually standard disinfection methods are used (Yildiz and Er, 2002; Huang et al., 2011). Here we present a comparative evaluation of disinfection strategies for hemp, and examine how treatment chemistry, seed provenance and cultivar influence the subsequent morphogenic competence of hypocotyl explants.

Water, sodium chloride and vinegar controls produced near-universal contamination or germination failure across all accessions, confirming that more stringent treatment is a prerequisite for axenic hemp seed culture. Among the treatments evaluated, a 75% ethanol baseline combined with sequential 1% H_2_O_2_ incubation performed broadly comparably to NaOCl-based protocols supplemented with Octave or PPM™. It is important point out that intrinsic germination failure of seedlings is difficult to distinguish from disinfection-induced decrease in germination rate. Hemp seed germination rate is lower on MS plates than on soil, thus a baseline comparison with soil is not informative. A baseline with non-disinfected seeds on MS plates is equally not feasible, because seedlings developing from non-disinfected seeds will have a much higher likelihood of contamination, therefore failing to germinate or progress into vegetative growth. While we did not observe adverse effects of the treatment such as necrosis or signs of oxidative stress in viable seedlings, we cannot exclude that our disinfection treatment is altering the plant physiology or causing adverse effects in the surviving seedlings due to phytotoxic effects. However, since germination rates observed for disinfected seeds germinated on soil were only slightly reduced as compared to untreated seeds germinated on soil, we consider phytotoxic effects of treatments largely negligible. A consistent finding across the entire dataset was the effect of seed provenance. In-house glasshouse-produced seeds achieved sterile seedling rates approximately over three times higher than commercially sourced material, and this difference was statistically significant and robust across accessions and treatment conditions. Commercially sourced seeds showed an overall average of ~10% disinfected seedling yield, but supplementation with Octave or PPM™ did lower contamination in some cases. While PPM is an established disinfection agent in plant cell culture (George and Tripepi, 2001), Octave is mostly used in industrial contexts as a fungizide (Denner et al., 1997; Henricot and Wedgwood, 2013) and is not typically used in plant cell culture. Here we demonstrate that it can be used for seed disinfection although further research regarding its effectiveness in hemp and possible applications for other species is needed. The provenance-driven difference likely reflects a higher and more variable microbial burden in field-produced, commercially or long-term stored hemp seeds relative to fresh seed material produced under controlled glasshouse conditions. Here, contamination load rather than disinfectant stringency appears to be the primary limiting factor for protocol success. Additionally, and not mutually exclusive, variations in cultivar used, storage duration and conditions, moisture content and seed maturity might significantly influence germination, regeneration and survival of seedlings, and are difficult to control for especially in commercially sourced seeds. The practical implication is direct: fresh, in-house produced seeds are strongly preferable for tissue culture work, and protocols benchmarked on laboratory-produced material should not be assumed to transfer to commercially sourced batches without re-validation.

With the developed protocol, we consistently were able to regenerate fertile hemp plants from hemp hypocotyl explants without hormone supplementation, confirming previously published observations (Long et al., 2022). Hemp is unusual in this hormone-free regeneration as approaches in other plant species usually require one or multiple plant growth regulators such as cytokinins or auxins for regeneration (Long et al., 2022). This regenerative capacity could be driven by endogenous hormone balance, explants maintaining juvenile tissue competence, or wound-induced organogenic signaling (Skoog and Miller, 1957; Sugimoto et al., 2010, 2019). The observed differences among cultivars indicate that genetic variation in intrinsic regeneration pathways may influence this hormone-independent response. Notably, we observed bipolar regeneration from hypocotyl explants, with shoots and roots developing from opposite ends of the same hypocotyl, which was previously reported as a side effect of hormone-free hypocotyl regeneration (Galán-Ávila et al., 2020). This observation suggests that a polarity remains within the hypocotyl and that cells within the hypocotyl retain totipotency, however, further research detailing the concrete mechanisms and possible specifics in hemp are required to complete our understanding of this process. The consistently high proportion of root-only regenerating explants across conditions suggests that supplementation with cytokinin warrants evaluation as a means of promoting hemp shoot organogenesis. The complete developmental cycle from disinfected hypocotyl explant through to fertile adult plant and viable seed was achieved, confirming that the disinfection conditions described here do not impair subsequent development or reproductive capacity. Further, we demonstrate that explantation and rooting of the newly generated shoot is not a required step in regeneration of hemp, which potentially lowers handling time and associated contamination risk. We also did not observe callus formation from hypocotyl explants under the described conditions, which is usually achieved by adding plant growth regulators (Hesami and Jones, 2021; Ahsan et al., 2024). For many applications, callus formation is not desirable and plant regeneration from hemp calli can be challenging, hence hormone-free regeneration might be a viable route in these cases.

Beyond their effect on contamination, disinfection treatments appeared to have a marked and independent influence on the morphogenic fate of hypocotyl explants. For in-house generated seeds of the hemp cultivar ‘Felina 32’, explants from seedlings previously treated with NaOCl combined with either Octave, or both Octave and PPM™, showed significantly higher rates of shoot and root co-regeneration on hormone-free medium compared to H_2_O_2_ or NaOCl alone. PPM™ supplementation also significantly reduced explant no regeneration relative to NaOCl alone. While these findings indicate an association between the disinfection treatments and morphogenic outcomes, they do not establish a direct pro-regenerative effect of Octave or PPM™. For example, it is possible that enhanced regeneration observed after treatment with Octave or PPM™ on hormone-free medium may reflect indirect effects of improved explant health or reduced microbial burden, which could preserve endogenous morphogenic competence, rather than a direct stimulation of regenerative pathways by these sterilizing agents. Notably, high concentrations of PPM™ have been reported as being phytotoxic and reducing regeneration rates (George and Tripepi, 2001), therefore optimizing for the lowest required concentration should be considered to further maximize the morphogenic outcomes. For commercially sourced seeds of the cultivar ‘Ferimon’, the pattern was reversed, with H_2_O_2_-treated explants showing the best regeneration outcomes. Hence, the relationship between disinfection treatment and morphogenic response could be source or even cultivar-dependent and should not be generalized from a single accession. Therefore, we recommend testing at least two different disinfection protocols when establishing the method for the first time in any given laboratory environment.

Plant regeneration from hypocotyl explants was achievable across all four accessions examined, demonstrating that this developmental capacity is broadly distributed within industrial hemp. Regeneration frequency and consistency varied substantially among accessions. ‘Fedora 17’ and ‘USO31’ showed the most favorable regeneration profiles, confirming previous findings (Galán-Ávila et al., 2020), with approximately one third of ‘Fedora 17’ explants achieving shoot and root co-regeneration, while ‘Kompolti’ and ‘Felina 32’ showed significantly lower rates of full regeneration under the same conditions. All accessions retained a high proportion of non-regenerating explants, and inter-accession variation in morphogenic trajectory is expected given the high heterozygosity and phenotypic diversity of industrial hemp Vergara et al., 2017, 2021; Kavanagh et al., 2025; Trubanová et al., 2026). Protocol optimization will therefore need to be at minimum accession-specific, and possibly batch-specific.

Taken together, these results support the following practical recommendations. Fresh, in-house produced seeds should be used wherever possible. Where commercially sourced material must be used, disinfection treatment with NaOCl combined with PPM™ and Octave should be considered. The protocol described here (**Figure 5**) provides a practical and documented starting framework that should be adapted based on seed source, batch history and intended downstream application.

**Figure 5:**
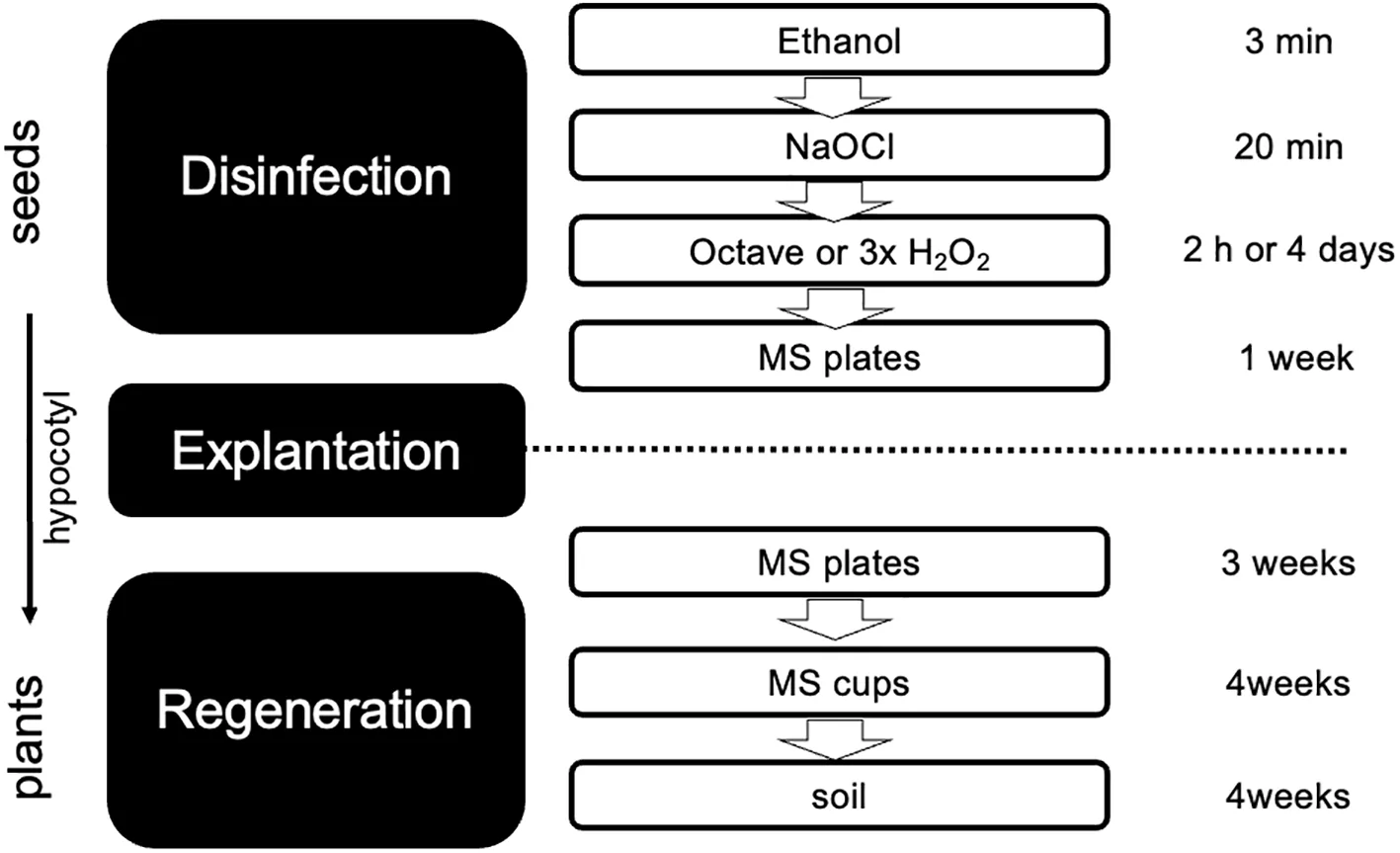
Protocol for disinfection, explantation and regeneration of *Cannabis sativa.* L. The workflow describes disinfection, explantation and regeneration from seed to seed via hypocotyl explants and regeneration.

## Data Availability

The original contributions presented in the study are included in the article/**Supplementary Material**. Further inquiries can be directed to the corresponding authors.

## Supplementary material

Supplementary Table 1 Disinfection treatment outcomes for in-house and commercial seeds.

Supplementary Table 2 Disinfection treated regeneration outcomes for in-house and commercial seeds.

Supplementary Figure 1 Surface disinfection efficacy of chemical and baseline treatments on hemp seeds.

Supplementary Figure 2 Effect of NaOCl-based disinfection treatments on hemp seeds of the cultivar ‘Ferimon’.

Supplementary Figure 3 Effect of different disinfection additives on the regeneration outcomes and viability of hemp hypocotyl explants of the cultivar ‘Ferimon’.
