# Supplementary material for: The viability gambit: an optimized disinfection protocol for industrial hemp (Cannabis sativa L.) seeds
Source: Front Plant Sci. 2026 Oct 13;17:1907649. doi: 10.3389/fpls.2026.1907649 (PMC13710263; doi:10.3389/fpls.2026.1907649)
Supplement: Supplementary file 1 [file DataSheet1.pdf]

Supplementary figures

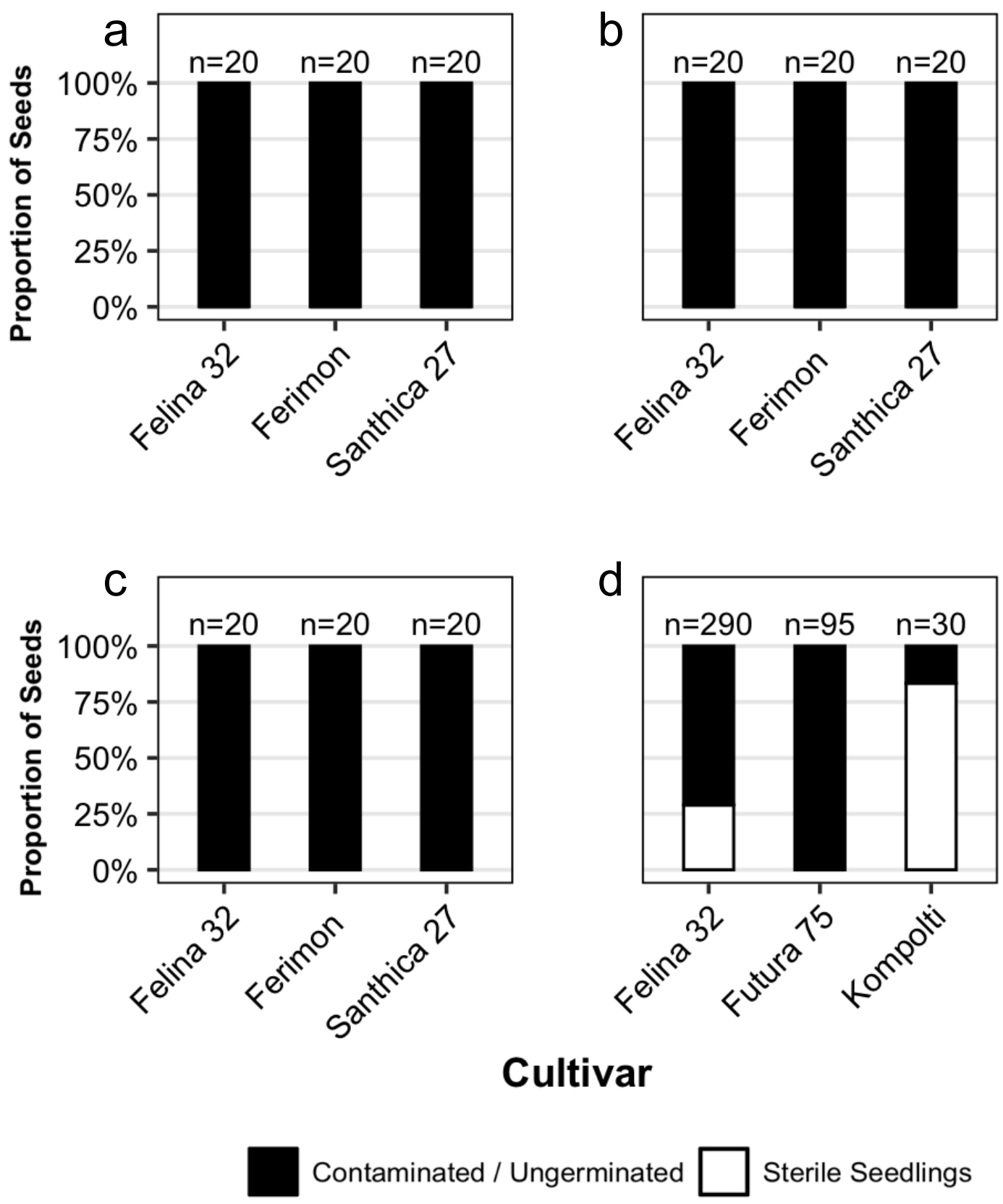

Figure S1. Surface sterilisation efficacy of chemical and baseline treatments on *Cannabis sativa*. L seeds. Water control (a) and basic treatments of seeds with NaCl (b) and vinegar (c) did not yield any germinated uncontaminated seedlings across all evaluated hemp cultivars. For the H_2O_2 treatment (d), sample sizes differ between cultivars because Felina 32, Futura 75 and Kompolti were each evaluated in a single experiment; an initial optimisation replicate of Felina 32 used to establish the H_2O_2 protocol (n=290) is included here and two other replicates were conducted at a different time point (Table S1). Though its efficacy was strongly influenced by cultivar (Fisher's exact test, ***, $p<0.001$). Stacked bar charts display the proportion of seeds yielding seedlings germinated uncontaminated seedlings (white bars) or exhibiting contamination/germination failure (black bars). Total sample sizes (n) contamination/germination failure (black bars). Total sample sizes (n) are displayed above each bar.

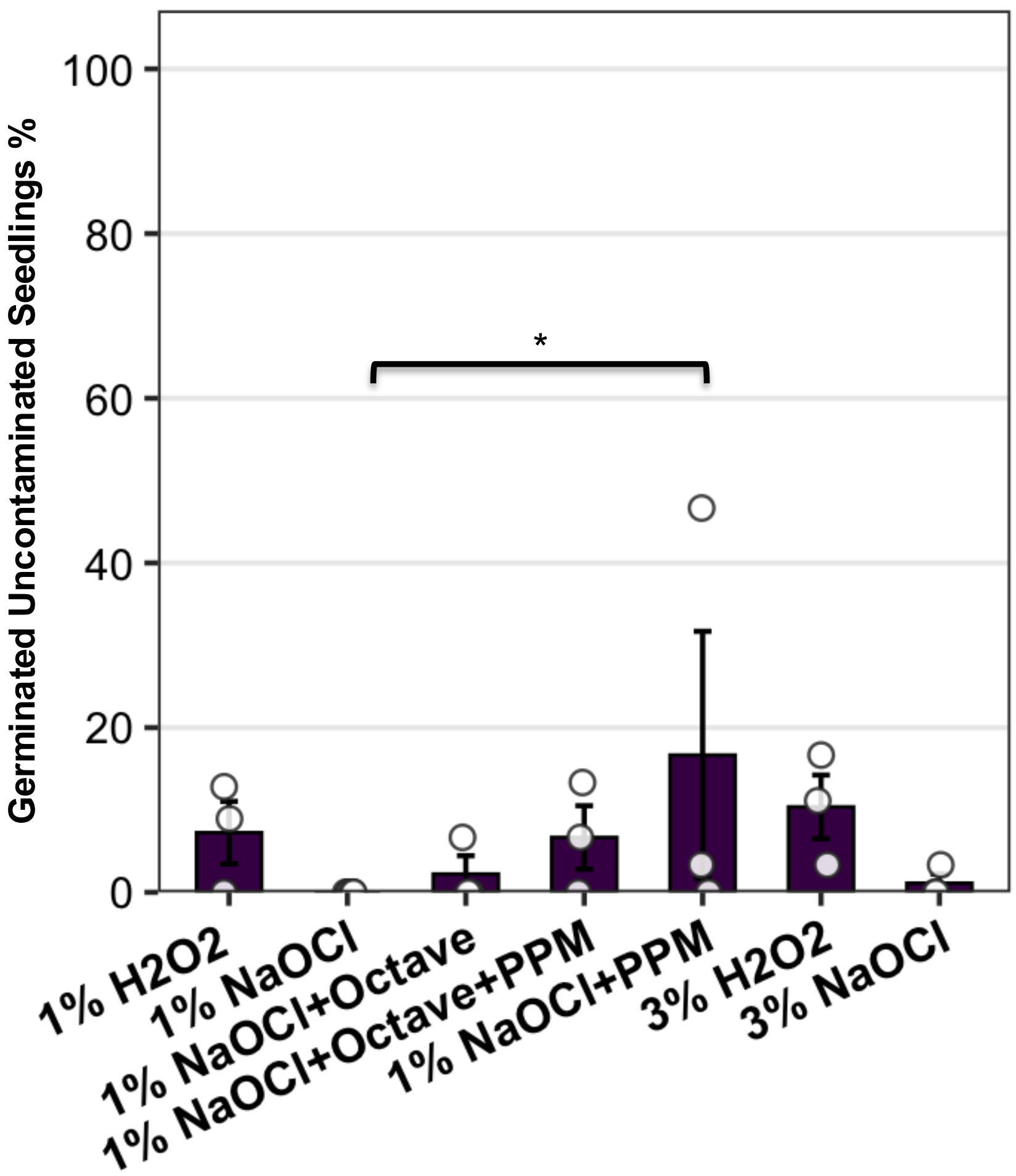

Figure S2. Effect of NaOCl-based disinfection treatments on *Cannabis sativa*. L seeds of the cultivar 'Ferimon'. Commercial hemp seeds of the cultivar 'Ferimon' were treated with different NaOCl disinfection protocols (1% NaOCl (n=184), 1% NaOCl and Octave (n=89), 1% NaOCl and PPM^{TM} (n=90), 1% NaOCl, Octave and PPM^{TM} (n=90), 1% H_2O_2 (n=125), 3% H_2O_2 (n=114), and 3% NaOCl (n=96)) and scored for contamination. Significant difference indicated with brackets (Tuckey's Pairwise comparison, *, $p<0.05$). Three independent biological replicates (n=3). Error bars indicate standard error.

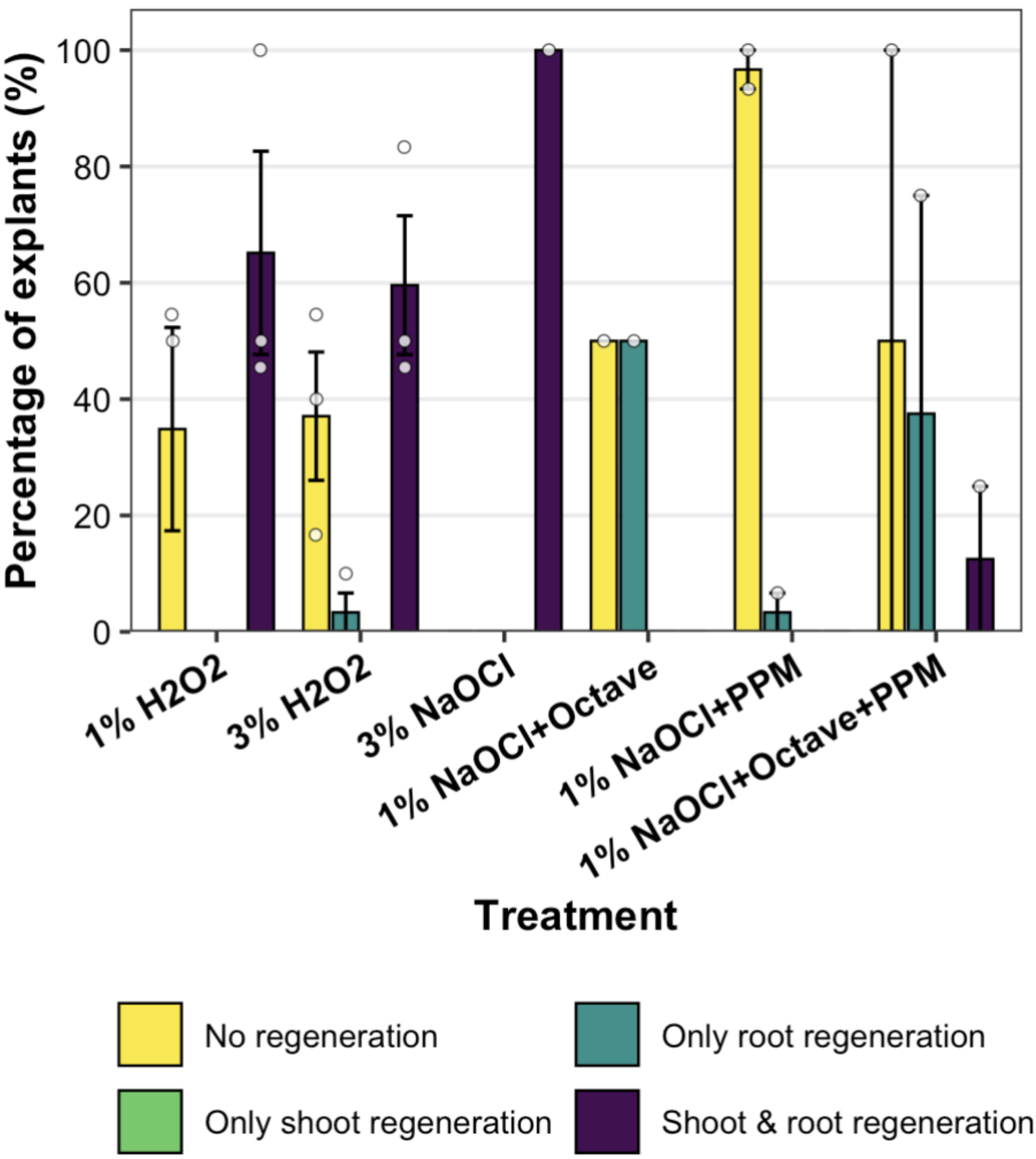

Figure S3. Effect of different disinfection additives on the regeneration outcomes and viability of hemp hypocotyl explants of the cultivar 'Ferimon'. Explants derived from seedlings of the hemp cultivar 'Ferimon' which underwent different disinfection protocols (1% NaOCl and Octave (n=2), 1% NaOCl and PPM^{TM} (n=16), 1% NaOCl, Octave and PPM^{TM} (n=5), 1% H_2O_2 (n=26), 3% H_2O_2 (n=27), and 3% NaOCl (n=7) and 1% NaOCl excluded due to no viable explants) were scored for their regeneration and categorized as no regeneration (yellow), only root regeneration (teal), only shoot regeneration (green) and shoot and root regeneration (dark blue). Here n refers to total number of explants.
